# Real-world use of pemigatinib for the treatment of cholangiocarcinoma in the US

**DOI:** 10.1093/oncolo/oyae204

**Published:** 2024-08-21

**Authors:** Kim Saverno, Kristin M Zimmerman Savill, Cherrishe Brown-Bickerstaff, Angele Kotomale, Michael Rodriguez, Bruce Feinberg, Haobo Ren, Mike Blecker, Richard Kim

**Affiliations:** Incyte Corporation, Wilmington, DE, United States; Cardinal Health, Real-World Evidence and Insights, Dublin, OH, United States; Cardinal Health, Real-World Evidence and Insights, Dublin, OH, United States; Cardinal Health, Real-World Evidence and Insights, Dublin, OH, United States; Incyte Corporation, Wilmington, DE, United States; Cardinal Health, Real-World Evidence and Insights, Dublin, OH, United States; Incyte Corporation, Wilmington, DE, United States; Incyte Corporation, Wilmington, DE, United States; Department of Gastrointestinal Oncology, Moffitt Cancer Center, Tampa, FL, United States

**Keywords:** cholangiocarcinoma, pemigatinib, FGFR2, real world

## Abstract

**Background:**

Pemigatinib demonstrated efficacy in *fibroblast growth factor receptor* (*FGFR*)-altered cholangiocarcinoma (CCA) in the FIGHT-202 trial. However, limited real-world evidence exists on treatment patterns and outcomes in this setting.

**Patients and Methods:**

Patient characteristics, treatment patterns, and outcomes of US adults who received pemigatinib for unresectable, locally advanced or metastatic CCA were collected via retrospective physician-abstracted chart review. Results were summarized using descriptive statistics.

**Results:**

Data from 120 patients (49.2% male; 55.0% White; 19.2% Hispanic; median age at initial pemigatinib prescription, 64.5 years) were collected from 18 physicians/practices. At the time of prescribing, 90.0% of patients had metastatic disease. *FGFR2* testing was completed for 92.5% of patients; of those, all but one (result unknown) tested positive, and 95.5% were tested using next-generation sequencing. Pemigatinib was prescribed as second- and third-line therapy among 94.2% and 5.8% of patients, respectively. The most common starting dosage was 13.5 mg daily for 14 days of 21-day cycles (87.5% of patients). Among 60 patients (50.0% of the full cohort) who discontinued pemigatinib during the 6.5-month median study follow-up period, 68.3% discontinued due to disease progression. The median real-world progression-free survival (rwPFS) from the date of pemigatinib initiation was 7.4 months (95% CI: 6.4-8.6), and the real-world overall response rate (rwORR) was 59.2% (95% CI: 50.0%-68.4%).

**Conclusion:**

This study complements the FIGHT-202 clinical trial by assessing the use of pemigatinib among a diverse population of patients with CCA under real-world conditions. Findings support the clinical benefit of pemigatinib demonstrated in FIGHT-202.

## Teaser

Although approved by the FDA as a targeted therapy for cholangiocarcinoma (CCA), little is known about the real-world use of pemigatinib in the US. This study assesses the real-world use of pemigatinib among US patients diagnosed with CCA.

**Implications for practice**

Since its 2020 approval as the first FDA-approved targeted therapy for cholangiocarcinoma (CCA), little remains known about the real-world use of pemigatinib in the US. This retrospective chart review included 120 US adults who received pemigatinib for CCA. Most patients (94.2%) received pemigatinib as second-line therapy for locally advanced or metastatic CCA. The median follow-up time was 6.5 months since initial pemigatinib prescription, and 50% were still receiving pemigatinib at last follow-up. The median real-world progression-free survival since pemigatinib initiation was 7.4 months. This real-world study complements the FIGHT-202 trial by assessing the use of pemigatinib among a diverse CCA population.

## Introduction

Cholangiocarcinoma (CCA) is a rare and aggressive cancer of the bile duct arising in the biliary epithelium.^1-4^ Disease classification is based on anatomical location in the biliary tract, with approximately 20% of cases classified as intrahepatic.^3^ For the majority of CCA cases (approximately 80%), disease is classified as extrahepatic, which can be further characterized as perihilar or distal, based on the tumor location in the biliary tract.^3^ In the US, CCA is diagnosed in approximately 8000 people annually and has a 5-year survival rate of <10%.^5^ CCA makes up 3% of all gastrointestinal tumors diagnosed in the US but accounts for approximately 20% of deaths.^6-8^ Patients with CCA typically have a poor prognosis due to late-stage diagnosis and the highly refractory nature of CCA in response to chemotherapy.^4,5,9^

Recent years have witnessed the advent of novel therapeutic options for CCA, including those that target actionable genetic alterations. Some of the most common genetic alterations observed in intrahepatic CCA are fibroblast growth factor receptor 2 (*FGFR2*) fusions or rearrangements, with an estimated incidence of 10%-15% among intrahepatic bile duct carcinomas.^10^*FGFR2* rearrangements are predominantly found in patients with intrahepatic CCA and infrequently among patients with extrahepatic disease.^2,11^ Findings from clinical trials have demonstrated that inhibition of the FGFR pathway may be an effective therapeutic option for certain patients with CCA.

Pemigatinib, a small-molecule kinase inhibitor that targets FGFR1, 2, and 3, received accelerated approval by the Food and Drug Administration (FDA) in April 2020 for the treatment of unresectable, locally advanced or metastatic CCA in previously treated adult patients with an *FGFR2* fusion or other rearrangement as detected by an FDA-approved test.^12,13^ This approval was supported by findings from the FIGHT-202 trial (NCT02924376), a phase II, open-label, multinational trial that explored the use of pemigatinib in previously treated unresectable, locally advanced or metastatic CCA patients with *FGFR2* fusions or rearrangements, other *FGF/FGFR* aberrations, or without *FGFR* alterations.^14^ There were a total of 107 patients in the study with *FGFR2* fusions or rearrangements (61% female, median age of 56 years, 74% White). The median duration of follow-up for the primary analysis of FIGHT-202 was 17.8 months (25th-75th percentile [p25-p75], 11.6-21.3).^15^ Among patients in the FIGHT-202 trial with *FGFR2* fusions or rearrangements, the centrally confirmed objective response rate for pemigatinib in the primary analysis was 35.5% (95% CI: 26.5-45.4), and the median duration of response was 7.5 months (95% CI: 5.7-14.5).^15^ The median overall survival (OS) was 21.1 months (95% CI: 14.8 to not estimable) and median progression-free survival was 6.9 months (95% CI: 6.2-9.6) among this patient cohort.^15^ Final analysis of FIGHT-202 occurred after a median (range) follow-up time of 45.4 months (95% CI: 19.9-53.7), and the results were largely consistent with the primary analysis.^14^

Since the approval of pemigatinib, additional *FGFR2* inhibitors have entered the US market in the CCA space. Futibatinib was approved by the FDA in 2022 for the treatment of previously treated, unresectable, locally advanced or metastatic intrahepatic CCA harboring *FGFR2* gene fusions or other rearrangements.^16^ Additionally, infigratinib was approved in 2021 as a second-line treatment option for patients with locally advanced or metastatic CCA harboring an *FGFR2* fusion or rearrangement,^17^ although it was later withdrawn from the market in 2023.^18^

While the therapeutic potential of pemigatinib has been demonstrated within the context of clinical trials, further research is needed to evaluate treatment patterns and clinical outcomes of pemigatinib in the real-world setting. The primary objectives of this study were to describe the characteristics, *FGFR2* testing patterns, and treatment patterns of patients treated with pemigatinib for locally advanced or metastatic CCA in the US as part of routine clinical care. This study also had an exploratory objective to describe the effectiveness outcomes of patients who received pemigatinib in this setting.

## Methods

### Data source and study design

This was a retrospective, observational, US multi-site, physician-abstracted medical chart review study of adults who received pemigatinib for unresectable, locally advanced or metastatic CCA. Medical oncologists from the Cardinal Health Oncology Provider Extended Network (OPEN) abstracted data from medical records of US patients who met prespecified eligibility criteria. OPEN is a geographically diverse, electronic medical record software/group purchasing organization-agnostic, US community of more than 7000 oncologists, hematologists, and urologists in predominantly community practices, including approximately 800 composing the real-world research community. Physicians were instructed to identify all eligible patients chronologically, starting with the earliest eligible patient who met the eligibility criteria during the index period and to select randomly from that point forward for up to 10 patients. Physicians provided information about their practice characteristics and abstracted de-identified data from medical records of eligible patients related to their demographics, clinical characteristics, biomarker testing patterns, treatment patterns, and clinical outcomes into a web-based electronic case report form (eCRF).

Extensive eCRF systems testing was conducted prior to the launch of data collection to ensure functionality across web-based user environments, looping logic to ensure proper alignment of data-related fields, and other automated and manual programmatic checks to reduce the possibility of collecting erroneous data. Additionally, the eCRF was subjected to user testing with 4 volunteer providers from OPEN to ensure functionality and that questions were interpreted correctly in relation to the data points of interest, as well as to determine the length of time needed for completion of data abstraction on a single patient. The testing simulated various clinical scenarios in which functional, editorial, and programmatic checks were performed in real time with the physicians. No data from the testing phase were used in the study.

Data abstraction for this study was conducted between February 3, 2023 and February 22, 2023. The study received approval and exemption for obtaining patient informed consent by a central Institutional Review Board and followed all standard research guidelines. In addition to real-time logic checks, data were subjected to clinical and scientific review for individual and aggregate validity. Data queries were resolved with the participating physicians directly, and any cases with data queries that could not be resolved were removed from the final analysis cohort. Physicians who did not receive a request to resolve a data query were subjected to random validation, in which they were required to re-enter certain data elements for a randomly selected eCRF that they had submitted. Inconsistency in data entered during validation and data originally entered would have resulted in exclusion of the physician and eCRFs that they had submitted from the study.

### Patient population

All patients included in this study had been prescribed pemigatinib for unresectable, locally advanced or metastatic CCA on or after April 17, 2020 and were at least 18 years of age when pemigatinib was initially prescribed. Patients were required to have at least 4 months of follow-up information from the time that pemigatinib was initially prescribed; however, otherwise eligible patients who had died during this 4-month period were also included. Patients were excluded if they received pemigatinib as part of an interventional trial, received systemic therapy for a primary malignancy other than cancer of unknown primary or hepatic cancer after initiation of pemigatinib or within the 12 months prior to date on which pemigatinib was initially prescribed, or if there was documentation or evidence that pemigatinib was prescribed to patient but never initiated by patient.

### Statistical analysis

Data were summarized using descriptive statistics. Time-to-event outcomes (ie, duration of therapy [DOT], rwPFS) were described using the Kaplan-Meier method, which accounts for right-censoring. Point estimates of rwPFS and real-world OS (rwOS) were reported at the 3- and 6-month timepoints. DOT was calculated using the initiation and discontinuation dates of pemigatinib with patients censored on the last encounter with the provider during therapy if the patient was still receiving pemigatinib at time of data abstraction. The pemigatinib rwORR was calculated as the sum of patients with reported real-world complete response (rwCR) and patients with real-world partial response (rwPR) as their reported best response to pemigatinib divided by all patients in the cohort. rwPFS was calculated as the time from pemigatinib initiation to time of physician-reported disease progression or death from any cause, whichever occurred first. Additionally, for patients with no documented progression event, a progression event was imputed at date of discontinuation if reason for discontinuation was reported as disease progression. Patients with no documented progression event were censored at the start date of new anticancer therapy if the reason for discontinuation was not reported as disease progression. Patients with no documented progression event or start date of new anticancer therapy were censored at the date of discontinuation or at date of last encounter if patient was still receiving pemigatinib.

All data processing and statistical analyses were performed using SAS v9.4.

## Results

### Physician and practice characteristics

Eighteen oncologists (Supplementary Table S1) abstracted patient data for this study. Among these physicians, nearly three-fourths (72.2%) reported being from private community practices ranging in size from small (2-5 physicians) to large (> 10 physicians). Participating oncologists had a median of 15.5 years (range 10-30 years) in practice. Over half (55.6%) of the physicians practiced in an urban setting, with the least frequently cited setting being rural (*n* = 1; 5.6%). All 4 US census regions were represented in terms of geographic location of their medical practice (33.3% from the Northeast, 27.8% from the West, 22.2% from the Midwest, and 16.7% from the South).

### Patient characteristics

A total of 120 patients were included in the study. Patient characteristics are presented in Table 1. Patients had a median age of 65.0 years (range: 37.0-83.0) at the time pemigatinib was initially prescribed, approximately half (50.8%) were female, just over half (55.0%) were White, and 19.2% were Hispanic. The majority (70.0%) had intrahepatic CCA at time of initial diagnosis, metastatic disease when pemigatinib was initially prescribed (baseline) (90.0%), and an Eastern Cooperative Oncology Group performance status (ECOG-PS) of 0-1 at baseline (78.3%). Of the CCA risk factors included in the eCRF, the most frequently cited CCA risk factors present at baseline among these patients were nonalcoholic fatty liver disease (NAFLD) among 12.5%, liver disease among 11.7%, primary sclerosing cholangitis among 3.3%, and hepatitis C among 3.3%. None of the patients had a history of hepatic cancer (data not shown). Among all patients, the most common sites of metastases at baseline were liver (64.2%) and lung (43.3%). The median follow-up time was 6.5 months (range, 1.7-21.7) since time at which pemigatinib was initially prescribed and 16.3 months (range, 5.7-77.2) since initial diagnosis with CCA (data not shown).

**Table 1.** Patient characteristics. Tabular representation of patient demographics and disease characteristics.

	All patients
	*N* = 120
Age at initial CCA diagnosis, median (range)	63.5 (36.0- 82.0)
Age at pemigatinib prescription, median (range)	65.0 (37.0- 83.0)
Sex at birth, female, *n(%)*	61 (50.8)
*Race* *, *n** *(%)*	
White	66 (55.0)
Black/African American	25 (20.8)
Other^a^	21 (17.5)
Unknown	8 (6.7)
*Ethnicity, n (%)*	
Hispanic	23 (19.2)
Non-Hispanic	94 (78.3)
*CCA location at initial diagnosis, n (%)*	
Intrahepatic	84 (70.0)
Extrahepatic (perihilar)	20 (16.7)
Extrahepatic (distal)	16 (13.3)
*CCA progression status at baseline, n (%)*	
Metastatic	108 (90.0)
Locally advanced	12 (10.0)
*ECOG-PS at baseline,* *n* *(%)* ^b^	
0-1	94 (78.3)
≥2	26 (21.7)
*Risk factors for CCA at the time of pemigatinib prescription, n (%)* ^b^ ^,^ ^c^	
NAFLD	15 (12.5)
Liver disease	14 (11.7)
PSC	4 (3.3)
Hepatitis C	4 (3.3)
Hepatitis B	3 (2.5)
Ulcerative colitis	3 (2.5)
Crohn’s disease	2 (1.7)
Cirrhosis	2 (1.7)
Hepatolithiasis	1 (0.8)
*Sites of metastases at baseline, n (%)* ^b^ ^,^ ^c^	
Liver	77 (64.2)
Lung	52 (43.3)
Regional/distal lymph node(s)	45 (37.5)
Local lymph node(s)	31 (25.8)
Peritoneum	24 (20.0)
Bone	16 (13.3)
Pleura	4 (3.3)
Brain	1 (0.8)
No sites of metastases	2 (1.7)

^a^Including Asian, Native Hawaiian or Other Pacific Islander, American Indian or Alaska Native, or mixed race.

^b^Baseline was defined as the time pemigatinib was prescribed.

^c^Not mutually exclusive.

Abbreviations: CCA, cholangiocarcinoma; ECOG-PS, Eastern Cooperative Oncology Group performance status; NAFLD, nonalcoholic fatty liver disease; PSC, primary sclerosing cholangitis.

### *FGFR2* testing patterns

*FGFR2* testing patterns are summarized in Table 2. Testing for *FGFR2* alterations was conducted for the majority of patients (92.5%, *n* = 111) and among these patients, all but one patient (result unknown) tested positive. Core-needle biopsy was used to gather samples for testing among 65.8% of the patients with *FGFR2* testing completed. Most (95.5%) received testing via next-generation sequencing, 2.7% via fluorescence in situ hybridization (FISH), and 1.8% via reverse transcription-polymerase chain reaction (RT-PCR).

**Table 2.** *FGFR2* testing patterns. Tabular representation of FGFR2 testing patterns and results of testing in patients who received pemigatinib.

	Patients with testing for *FGFR2* alterations completed
	*N* = 111
*Results of testing, n (%)*	
Fusion rearrangement	102 (91.9)
* Fusion partner*	
* BICC1*	38 (37.3)
* AHCYL1*	10 (9.8)
* CCDC6*	9 (8.8)
* KIAA1217*	7 (6.9)
* TACC2*	4 (3.9)
Unknown	34 (33.3)
Positive (for FISH testing)	3 (2.7)
Gene amplification	3 (2.7)
Activating mutation	2 (1.8)
Overexpression	0 (0)
Negative	0 (0)
Unknown	1 (0.9)
*Biopsy technique(s) used to gather FGFR2 alteration testing tissue sample, n (%)* ^a^	
Core-needle biopsy	73 (65.8)
CT/MRI guided	39 (35.1)
Surgical resection	7 (6.3)
Fine-needle aspiration	6 (5.4)
Brush cytology	2 (1.8)
*Testing completed prior to time of initial pemigatinib prescription*, *n(%)*	110 (99.1)
*Time from* *FGFR2* *alteration testing to initial pemigatinib prescription, median (range), months*	5.0 (0.1-23.7)
*Testing method used, n (%)*	
NGS	106 (95.5)
FISH	3 (2.7)
RT-PCR	2 (1.8)

^a^Not mutually exclusive, hence does not total to 100%.

Abbreviations: *BICC1*, *BicC family RNA-binding protein 1*; CT, computed tomography; *FGFR2*, fibroblast growth factor receptor 2; FISH, fluorescence in situ hybridization; MRI, magnetic resonance imaging; NGS, next-generation sequencing.

Of the patients who were tested for *FGFR2* alterations, *FGFR2* fusion rearrangements were detected in samples from 91.9%. The most frequently reported *FGFR2* fusion partner was *BicC family RNA-binding protein 1* (*BICC1*), detected among 37.3% of patients with fusion rearrangements. For all but one patient who had *FGFR2* testing, the testing was conducted prior to when pemigatinib was initially prescribed; among these patients, the median time from testing to initial pemigatinib prescription was 5.0 months [range, 0.1-23.7]).

### Treatment and utilization patterns

Treatment patterns are described in Table 3 and Supplementary Figure S1. Prior to diagnosis with advanced/metastatic disease, none of the patients were reported to have received neoadjuvant systemic therapy or radiation, approximately 12.5% had received surgery, and approximately 3.3% had received adjuvant systemic therapy (capecitabine for *n* = 2 patients, gemcitabine + oxaliplatin for *n* = 1 patient, and gemcitabine + capecitabine for *n* = 1 patient). Among all patients, the most commonly received therapies in the first-line setting were cisplatin combined with gemcitabine (60.8%) followed by cisplatin plus gemcitabine and durvalumab (15.0%). Overall, the therapies received in the first-line setting can be broadly classified as chemotherapy (94.2%), immunotherapy (18.3%), and targeted therapy (2.5%). Among all patients, the median DOT on first-line therapy was 4.9 months (95% CI: 4.4-5.7). Most (94.2%) patients received pemigatinib in the second-line therapy for locally advanced or metastatic CCA, and 5.8% received it in the third-line setting. Among those patients who received pemigatinib in the third-line setting (*n* = 7), the second-line therapies consisted of 5-fluorouracil + oxaliplatin (71.4%), cisplatin + gemcitabine (14.3%), or pembrolizumab (14.3%). No patients in this study had sequential use of *FGFR2* inhibitors.

**Table 3.** Pemigatinib treatment patterns. Tabular representation of patterns of pemigatinib treatment in patients.

	All patients
	*N* = 120
*Pemigatinib dose/frequency at initial prescription, n (%)*	
13.5 mg	107 (89.2)
Daily × 14 days on and repeat every 21 days	105 (87.5)
Daily × 10 days on and repeat every 21 days	2 (1.7)
9.0 mg	10 (8.3)
Daily × 14 days on and repeat every 21 days	6 (5.0)
Daily × 21 days on and repeat every 28 days	2 (1.7)
Daily (continuously with no breaks)	1 (0.8)
Daily × 14 days on and repeat every 28 days	1 (0.8)
4.5 mg	3 (2.5)
Every other day × 21 days and repeat every 28 days	2 (1.7)
Daily × 14 days on and repeat every 28 days	1 (0.8)
*Line of therapy for locally advanced/metastatic CCA in which pemigatinib was received*	
Pemigatinib received as second-line therapy, *n* (%)	113 (94.2)
* Prior treatment received as first-line therapy, n (% patients who received pemigatinib as second line)* ^a^	
Chemotherapy	107 (94.7)
Immunotherapy	18 (15.9)
Targeted therapy	3 (2.7)
Pemigatinib received as third-line therapy, *n* (%)	7 (5.8)
* Prior treatment received as second-line therapy, n (% patients who received pemigatinib as third line)*	
Chemotherapy	6 (85.7)
Immunotherapy	4 (57.1)
*Pemigatinib therapy status at time of data collection*	
Still on pemigatinib, *n* (%)	60 (50.0)
Discontinued pemigatinib, *n* (%)	60 (50.0)
* Primary rationale for discontinuation, n (% who discontinued)*	
Disease progression (confirmed with scan)	39 (65.0)
Patient choice	7 (11.7)
Death	6 (10.0)
Toxicity	3 (5.0)
Disease progression (defined clinically)	2 (3.3)
Scheduled duration of therapy complete	1 (1.7)
Other	2 (3.3)
*Pemigatinib duration of therapy* ^b^	
Censored observations, *n* (%)	60 (50)
Events, *n* (%)	60 (50)
Median (95% CI), months	7.4 (6.2-8.8)

^a^Not mutually exclusive, hence does not total to 100%.

^b^Time from the initiation to discontinuation of pemigatinib. Patients still receiving pemigatinib at the last encounter were censored on the date of last encounter.

Abbreviation: CCA, cholangiocarcinoma.

The majority of patients (87.5%) were initially prescribed pemigatinib at a dose of 13.5 mg daily for 14 days, repeating every 21 days, consistent with the FDA-approved prescribing information.^13^ The second most frequent starting dose was 9.0 mg daily for 14 days, repeating every 21 days (among 5.0%). A total of 3 patients initiated pemigatinib with the 4.5 mg strength tablet. During the 6.5-month median follow-up period, pemigatinib dose or frequency reductions were experienced by 12.5% of patients while dose interruptions were experienced by 2.5% (data not shown). Half of all patients had discontinued pemigatinib at the time of data abstraction with the most common rationale for discontinuation (65%) being disease progression as confirmed by scan. Among all patients, the median DOT with pemigatinib was 7.4 months (95% CI: 6.2-8.8).

### Exploratory clinical outcomes

**Figure 1.**
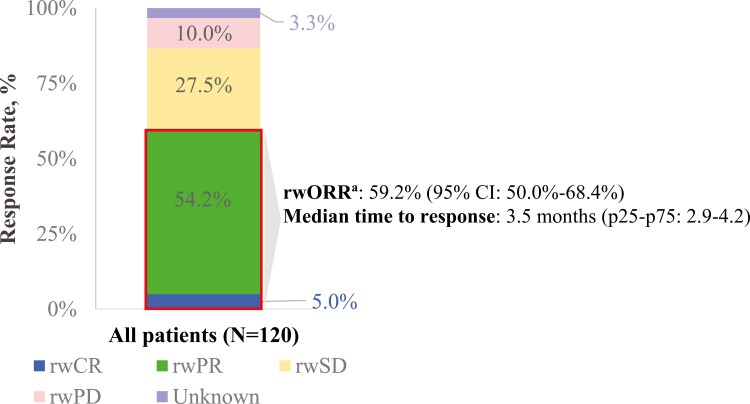
Best response to pemigatinib. ^a^The sum of patients with reported real-world complete response and patients with real-world partial response as their reported best response to pemigatinib divided by all patients in the cohort. Abbreviations: p25-p75, 25th-75th percentile; rwCR, real-world complete response; rwORR, real-world overall response rate; rwPD, real-world progressive disease; rwPR, real-world partial response; rwSD, real-world stable disease.

**Figure 2.**
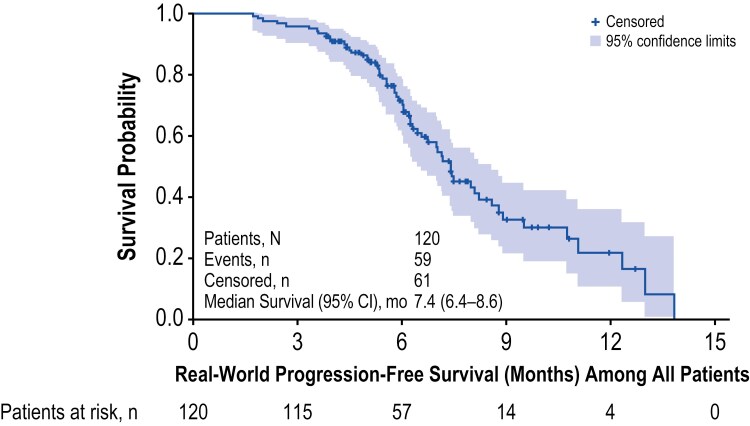
rwPFS for Pemigatinib. rwPFS, real-world progression-free survival.

Clinical outcomes are summarized in Table 4 and Figures 1 and 2. Disease response to pemigatinib was available for most patients in the study (96.7%, *n* = 116). The rwORR for pemigatinib was 59.2% (95% CI: 50.0%-68.4%), with 5.0% achieving an rwCR and 54.2% achieving an rwPR. The median time to response among those achieving a complete or partial response to pemigatinib was 3.5 months (p25-p75: 2.9-4.2). Among all patients, 59.2% were still alive at the time of data collection. The median rwPFS for pemigatinib was 7.4 months (95% CI: 6.4-8.6). The rwPFS proportion was 95.8% (95% CI: 90.3-98.2) at 3 months post-pemigatinib initiation and 71.5% (95% CI: 61.4-79.4) at 6 months post initiation. The rwOS proportion was 95.8% (95% CI: 90.3-98.2) at 3 months and 88.4% (95% CI: 80.3-93.3) at 6 months following pemigatinib initiation.

**Table 4.** Pemigatinib clinical outcomes. Tabular representation of best real-world clinical outcomes for patients treated with pemigatinib.

	All patients
	*N* = 120
Patient alive at data abstraction, *n* (%)	71 (59.2)
Best response available, *n* (%)	116 (96.7)
rwORR, *n* (%) (95% CI)^a^	71 (59.2) (50.0-68.4)
*Best response*	
rwCR, *n* (%) (95% CI)	6 (5.0) (1.8-12.9)
rwPR, *n* (%) (95% CI)	65 (54.2) (42.5-65.4)
rwSD, *n* (%) (95% CI)	33 (27.5) (18.4-39.0)
rwPD, *n* (%) (95% CI)	12 (10.0) (4.9-19.3)
Unknown, *n* (%) (95% CI)	4 (3.3) (1.0-10.6)
*Imaging used for best response, n (%)* ^b^	
CT	78 (67.2)
PET/CT	35 (30.2)
PET/MRI	1 (0.9)
PET	1 (0.9)
MRI	1 (0.9)
Bone scan	0 (0.0)
*KM-estimated rwPFS^c^*	
Censored observations, *n* (%)	61 (50.8)
Events, *n* (%)	59 (49.2)
Median (95% CI), months	7.4 (6.4-8.6)
*Point estimates for rwPFS*	
3 months	
Number at risk	115
Point estimate (%) (95% CI)	95.8 (90.3-98.2)
6 months	
Number at risk	57
Point estimate (%) (95% CI)	71.5 (61.4-79.4)
*Point estimates for rwOS*	
3 months	
Number at risk	115
Point estimate (%) (95% CI)	95.8 (90.3-98.2)
6 months	
Number at risk	73
Point estimate (%) (95% CI)	88.4 (80.3-93.3)

^a^The sum of patients with reported real-world complete response and patients with real-world partial response as their reported best response to pemigatinib divided by the number of patients in the entire cohort.

^b^Not mutually exclusive, hence does not total to 100%.

^c^rwPFS was calculated as the time from pemigatinib initiation to date of physician-reported disease progression or death from any cause, whichever occurs first. Additionally, for patients with no documented progression event, a progression event was imputed at date of discontinuation if reason for discontinuation was reported as disease progression. Patients with no documented progression event were censored at start date of new anti-cancer therapy if reason for discontinuation was not reported as disease progression. Patients with no documented progression event or start date of new anti-cancer therapy were censored at date of discontinuation or at date of last encounter if patient was still receiving pemigatinib.

Abbreviations: CT, computed tomography; KM, Kaplan-Meier; MRI, magnetic resonance imaging; PET, positron emission tomography, rwCR, real-world complete response; rwORR, real-world overall response rate; rwPD, real-world progressive disease; rwPFS, real-world progression-free survival; rwPR, real-world partial response; rwSD, real-world stable disease.

## Discussion

Findings from this retrospective, primarily community oncology-based physician-abstracted medical chart review study demonstrate the demographics, clinical characteristics, *FGFR2* testing patterns, treatment patterns, and clinical outcomes of 120 patients treated with pemigatinib for unresectable, locally advanced or metastatic CCA as a part of routine clinical care. Given the scarcity of real-world evidence in this setting, the findings from this study represent an important advancement in the knowledge of real-world utilization of and outcomes associated with pemigatinib for the treatment of unresectable, locally advanced or metastatic CCA.

Importantly, the US real-world population in this study was racially and ethnically diverse. White patients made up slightly more than half of the total study population, with the remaining being non-White or having unknown race (approximately 7%). Additionally, Hispanic patients, a typically underrepresented population, made up just under one-fifth of patients in this study. Hence, this study provides findings related to pemigatinib treatment patterns and outcomes among a population with particularly limited data. The diversity of this real-world population is aligned with or even more diverse than the published racial and ethnic distribution of the broader US CCA population, among whom approximately 78% are White patients and 7.8% are of Hispanic ethnicity.^19,20^ Further, our real-world study population was more racially diverse than the *FGFR2* fusion/rearrangement positive cohort of FIGHT-202 (74% White).^14,15^ Of note, males and females were nearly equally represented in these data, and the study population included patients between 37 and 83 years of age at pemigatinib prescription (median age at initial CCA diagnosis, 63.5 years). While the distribution in terms of age was aligned with published findings from the broader US CCA population^20^ and real-world analysis of US patients with *FGFR2* fusions/rearrangements and advanced CCA, (median age at initial diagnosis, 63 years),^11^ the proportion of females was lower in our real-world study than that published in other real-world analyses of patients with CCA with *FGFR2* genetic aberrations (50.8% vs approximately 63%).^21,22^

One of the unexpected findings of this study was the relatively high percentage of patients with extrahepatic disease (distal or perihilar) at time of initial CCA diagnosis. While a previously reported US real-world analysis of patients indicated that 95% of patients with an *FGFR2* fusion/rearrangement had intrahepatic disease,^11^ and 98% of patients in FIGHT-202 with *FGFR2* fusion had intrahepatic disease,^11^ participating physicians in our real-world study reported that 70.0% of the patients who received pemigatinib had intrahepatic CCA at initial CCA diagnosis. Of the 102 patients in our study with *FGFR2* fusions reported, 25.5% had extrahepatic CCA. In considering possible reasons for this difference in the proportion of intrahepatic CCA between studies, it is feasible that the care setting may play a role. Additional follow-up with participating physicians in this study regarding the lower-than-expected proportion of patients with intrahepatic disease in this sample supported this notion. Typically, intrahepatic CCA presents as a large mass because the tumor does not cause clinical symptoms in its early stages, whereas extrahepatic CCA is typically small at the time of presentation because the bile ducts are occluded in its early stage, and patients present with jaundice.^23^ Obstructive jaundice is more likely a community oncologic referral whereas asymptomatic hepatic mass is more likely tertiary care center referral for resection. Community oncologists may see disproportionately more extrahepatic disease, and therefore their distribution of patients with *FGFR* aberration positive CCA may have greater extrahepatic representation. The variance in extrahepatic designation warrants additional analysis to understand if it is related to differences in community versus academic site referral patterns, mislabeling by treating physicians, a combination thereof, or other factors.

The recommended starting dose for pemigatinib, and dose administered in the FIGHT-202 trial, is 13.5 mg once daily for 14 days followed by 7 days off in 21-day cycles.^13-15^ Just under 90% of patients in this study received pemigatinib according to this starting dose and schedule, suggesting that oncologists are generally utilizing pemigatinib according to label guidelines in terms of starting dosage and frequency. Interestingly, a small proportion of patients in this study (*n* = 9, 7.5%) received pemigatinib despite the fact that *FGFR2* testing was not reported to have been conducted. Among patients in this study with *FGFR2* testing conducted, approximately 95% had *FGFR2* gene fusion rearrangements or had positive *FGFR2* FISH testing results, with 6 patients having received pemigatinib when *FGFR2* testing results were unknown or an activating mutation or gene amplification was detected. Altogether, these observations suggest that for a small proportion of patients, oncologists may be prescribing pemigatinib inconsistently with its approved label, but, generally, pemigatinib real-world utilization is in alignment with the way it was approved by the FDA. Importantly, pemigatinib was received as second-line therapy for the majority of patients in this real-world US population, with no sequential use of *FGFR2* inhibitors and no front-line use.

Pemigatinib dose reductions and dose interruptions were experienced by 12.5% and 2.5%, respectively, of the study population, in contrast to 14% and 42%, respectively, of the overall FIGHT-202 trial popluation.^14,15^ Multiple factors may be driving these differences between studies. First, the median follow-up time since pemigatinib initiation was much shorter for our real-world study in comparison to the primary analysis of the FIGHT-202 trial (6.3 vs 17.8 months), and half of patients in our real-world study were still receiving pemigatinib at the time of last follow-up. Hence, there was less time to observe dose reductions or treatment interruptions in this real-world study. Secondly, real-world clinical trial protocols often employ monitoring schedules and dose reduction criteria that are more stringent than what is observed in the real-world setting, which may lead to more dose reductions and interruptions than what is observed as part of routine clinical care. Additionally, a proportion of patients in the real-world population received pemigatinib at doses lower than the 13.5 mg dose used in FIGHT-202, perhaps lessening the need of further dose reductions. Lastly, the physicians who participated in this study appeared to be more frequent prescribers of pemigatinib and had likely gained familiarity with the drug, therefore also contributing to less frequent dose adjustments than observed in the FIGHT-202 primary analysis.

Given the pemigatinib rwORR of 59.2% (54.2% with a rwPR and 5.0% with a rwCR) and median rwPFS of 7.4 months observed in this study, these real-world study outcomes data support the clinical benefit of pemigatinib in this indication and setting. These data can be considered in the context of findings from the patients in the FIGHT-202 clinical trial with *FGFR2* fusions or rearrangements. However, differences between the trial and this US medical chart review in terms of the patient populations and care settings should be considered. Of note, nearly three-fifths of the real-world study population was still alive at data collection. However, the median rwPFS observed in this real-world study was similar to that observed among patients in the FIGHT-202 clinical trial with *FGFR2* fusions or rearrangements (7.4 and 6.9 months, respectively).^14^ While the rwORR observed was higher than that of patients in the FIGHT-202 clinical trial with *FGFR2* fusions or rearrangements (59.2% vs 35.5%), disease response assessment may differ in routine clinical care in comparison to the clinical trial setting. Disease response assessment in real-world settings is performed at variable frequencies, using a variety of different diagnostic modalities, at differing sites of care, and interpreted by different radiologists.^24^

### Limitations

A limited number of physicians (*N* = 18) participated in this study, who were largely community based and seemed to have substantial experience with pemigatinib given that participating physicians reported having an average of 13 patients who met study eligibility criteria. Treatment patterns of physicians outside of this sample have not been captured in this study. Observed treatment sequences may not be reflective of current treatment practices due to the rapidly changing treatment landscape in CCA. Additionally, the median follow-up time of 6.5 months since initiation with pemigatinib was short; half of patients included in this study were still receiving pemigatinib at the last encounter with the abstracting physician or their clinical practice. This limits the ability to better understand long-term outcomes of patients treated with pemigatinib or treatment sequencing post-pemigatinib. It is also currently unclear as to whether the findings of this study are representative of the larger US population with CCA receiving pemigatinib for unresectable, locally advanced or metastatic disease.

## Conclusion

This study complements the FIGHT-202 clinical trial by assessing the use of pemigatinib among a more diverse population of patients with CCA under real-world conditions. Secondarily, the rwORR and rwPFS from this study support the clinical benefit of pemigatinib demonstrated in FIGHT-202. Further real-world evidence is warranted to better understand pemigatinib utilization and associated outcomes when used for the treatment of locally advanced or metastatic CCA.

## Supplementary material

Supplementary material is available at *The Oncologist* online.

oyae204_suppl_Supplementary_TableS1

oyae204_suppl_Supplementary_FigureS1

## Data Availability

Incyte Corporation (Wilmington, DE, USA) is committed to data sharing that advances science and medicine while protecting patient privacy. Qualified external scientific researchers may request anonymized datasets owned by Incyte for the purpose of conducting legitimate scientific research. Researchers may request anonymized datasets from any interventional study (except Phase I studies) for which the product and indication have been approved on or after 1 January 2020 in at least one major market (eg. US, EU, JPN). Data will be available for request after the primary publication or 2 years after the study has ended. Information on Incyte’s clinical trial data sharing policy and instructions for submitting clinical trial data requests are available at: https://www.incyte.com/Portals/0/Assets/Compliance%20and%20Transparency/clinical-trial-data-sharing.pdf?ver=2020-05-21-132838-960.
