# Supplementary material for: Real-world use of pemigatinib for the treatment of cholangiocarcinoma in the US
Source: Oncologist. 2024 Aug 21;30(1):oyae204. doi: 10.1093/oncolo/oyae204 (PMC11783287; doi:10.1093/oncolo/oyae204)
Supplement: oyae204_suppl_Supplementary_TableS1 [file oyae204_suppl_supplementary_tables1.pdf]

Supplementary Table 1. Physician and Practice Characteristics

	**All providers N = 18**
**Primary practice setting, n (%)**	
Solo practitioner	0 (0)
Small private community practice (2-5 physicians)	2 (11.1)
Medium-sized private community practice (6-10 physicians)	5 (27.8)
Large private community practice (>10 physicians)	6 (33.3)
Community practice owned by an academic center	0 (0)
Academic medical center	4 (22.2)
Affiliated teaching hospital	1 (5.5)
VA/military hospital/DoD	0 (0)
Other, please specify	0 (0)
**Provider number of years in practice, n (%)**	
Mean, STD	17.8, 6.6
Median, 25P-75P	15.5, 12.0-20.0
Min, Max	10, 30
**Practice urbanicity, n (%)**	
Urban	10 (55.6)
Suburban	7 (38.9)
Rural	1 (5.6)
**Geographic location, n (%)**	
Northeast	6 (33.3)
Midwest	4 (22.2)
South	3 (16.7)
West	5 (27.8)

Abbreviations: DoD, Department of Defense; 25P-75P, 25th-75th percentile; max, maximum; min, minimum; STD, standard deviation; VA, Veterans Affairs
