# Supplementary material for: Real-world use of pemigatinib for the treatment of cholangiocarcinoma in the US
Source: Oncologist. 2024 Aug 21;30(1):oyae204. doi: 10.1093/oncolo/oyae204 (PMC11783287; doi:10.1093/oncolo/oyae204)
Supplement: oyae204_suppl_Supplementary_FigureS1 [file oyae204_suppl_supplementary_figures1.pdf]

Supplementary Figure 1: Regimens Received as First-Line Therapy for Advanced/Metastatic CCA

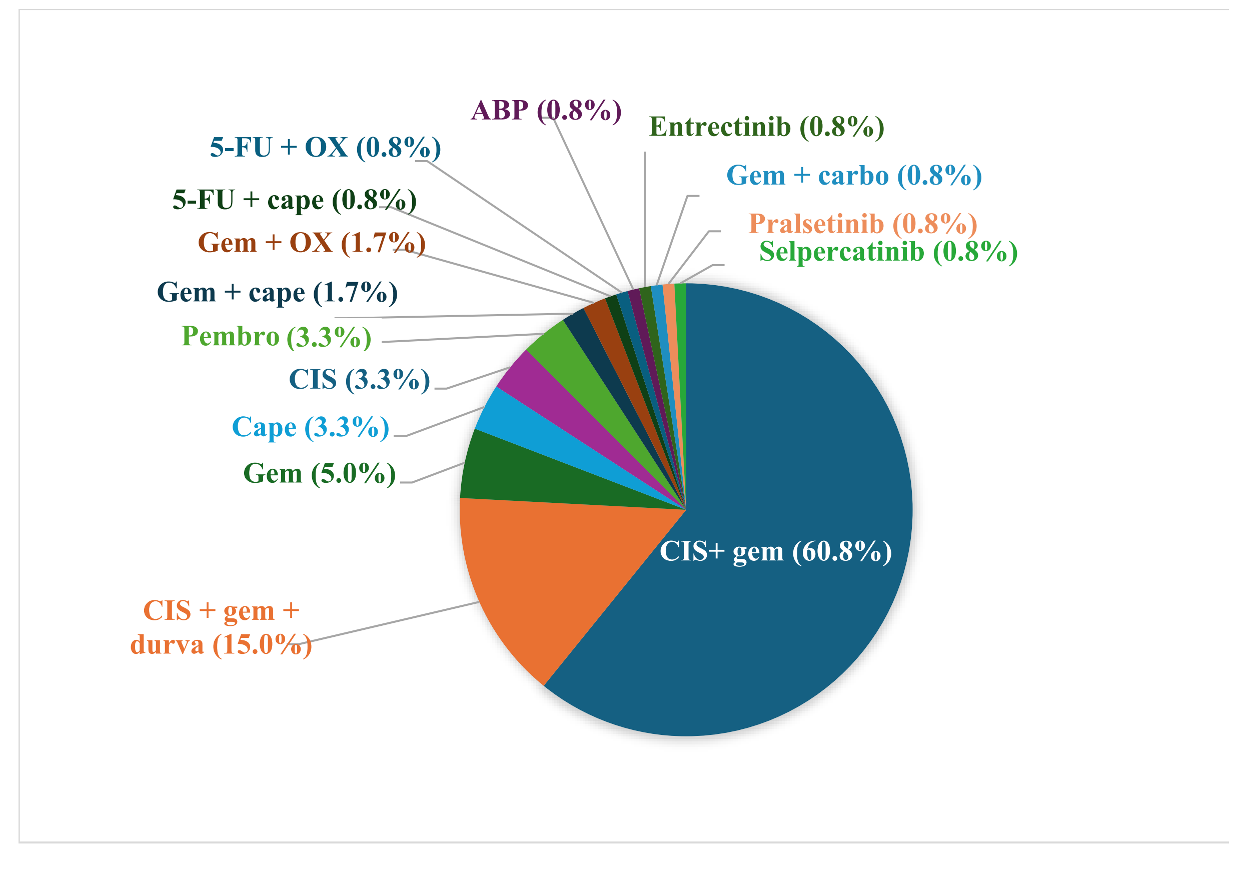

Abbreviations: 5-FU, 5-fluorouracil; ABP, albumin-bound paclitaxel; cape, capecitabine; carbo, carboplatin; CCA, cholangiocarcinoma; CIS, cisplatin; durva, durvalumab; gem, gemcitabine; OX, oxaliplatin; pembro, pembrolizumab
